# Association of Pulmonary Septic Embolism and Pulmonary Arterial Thromboembolism in Lemierre Syndrome: A Case Report and Literature Review

**DOI:** 10.1002/rcr2.70266

**Published:** 2025-07-15

**Authors:** Antonio Fabozzi, Alessandro Siena, Alessia Steffanina, Silvia Iannuzzi, Matteo Bonini, Paolo Palange

**Affiliations:** ^1^ Department of Public Health and Infectious Diseases, Pulmonology Unit, Policlinico Umberto I “Sapienza” University of Rome Rome Italy

**Keywords:** infections, Lemierre syndrome, multiple pulmonary nodules, pulmonary embolism, thrombophlebitis

## Abstract

Lemierre syndrome (LS) is a rapidly progressing disease characterised by a recent oropharyngeal infection, complicated by septic emboli and thrombophlebitis of the internal jugular vein (IJV). We describe a case of a 65‐year‐old woman who presented to the emergency room with a 14‐day history of progressive occipital headache, fever and odynophagia. Radiological investigations showed bilateral IJV thrombophlebitis, multiple cavitated pulmonary nodules suggestive of septic emboli and a subsegmental pulmonary arterial thromboembolism. A diagnosis of LS was made. The patient received antibiotic treatment with intravenous ceftriaxone and clindamycin for 2 weeks, followed by 4 weeks of oral clindamycin at home and anticoagulation with enoxaparin followed by warfarin. One‐month follow‐up imaging revealed complete resolution of IJV thrombosis and pulmonary findings. This case displayed the rarely reported association of pulmonary arterial thromboembolism and pulmonary septic emboli.

## Graphical abstract

We report a rare presentation of Lemierre's syndrome in a 65‐year‐old woman, characterised by the coexistence of septic pulmonary emboli and pulmonary arterial thromboembolism, both documented on CT pulmonary angiography.

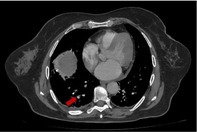

## 1 Introduction

Lemierre syndrome (LS) is defined by a recent oropharyngeal infection history, complicated by septicaemia with septic emboli and thrombophlebitis of the internal jugular vein (IJV) [1]. *Fusobacterium necrophorum* (FN) is considered the most frequently implicated pathogen, isolated by culture from 68% to 51% of cases in accordance with current data available [2, 3]. There are limited epidemiological data on LS, but recent studies estimate an overall incidence between 0.5 and 5.5 cases per million [4, 5]. Initial clinical manifestations include pharyngodynia, neck pain, odynophagia and sometimes fever [6, 7]. The second phase of the disease, which occurs a few days or weeks after, is characterised by IJV thrombophlebitis and sepsis [8]. The dissemination of septic emboli in other organs is also common and occurs primarily in the lungs, where complications such as empyema or pleural effusion can also occur [9]. Other possible sites where septic emboli can spread include the liver, brain and spleen [4]. The generally accepted diagnostic criteria for LS are three: I. a recent history of pharyngeal illness; 2. Evidence of septic emboli; 3. IJV thrombophlebitis or FN isolated by blood culture [10]. Antibiotic therapy should cover anaerobic pathogens and be tailored to the identified microbiological agent when isolated [8]. Commonly used antibiotics include clindamycin, penicillins and metronidazole [11]. Intravenous administration for the first weeks is strongly recommended, followed by oral therapy, with a total duration of three to five weeks [2, 9].

## 2 Case Report

**FIGURE 1:**
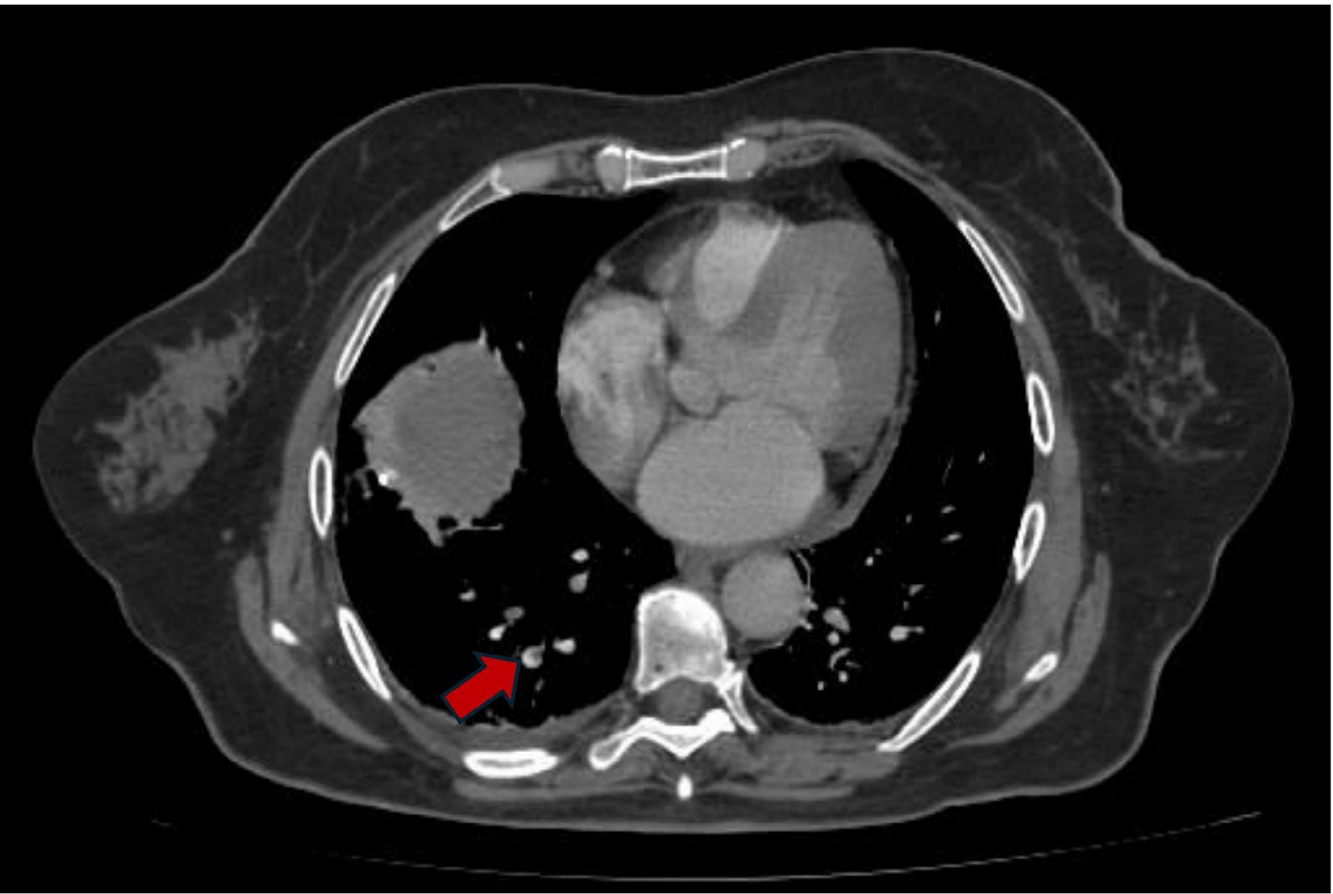
CT pulmonary angiography showed thrombotic appositions in the lower right lobar pulmonary artery (red flag).

**FIGURE 2:**
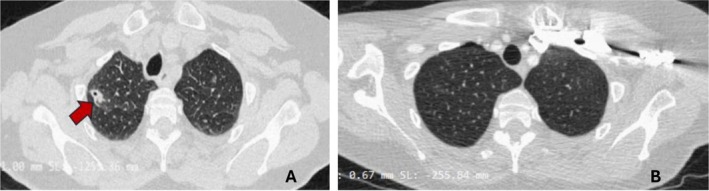
(A): Chest CT showed a partially cavitated nodule in the right upper lobe (red flag). (B): Complete regression of the upper right lobe nodule at 1 month follow‐up chest CT.

We report the case of a 65‐year‐old woman who presented to the emergency department with a 14‐days history of progressive occipitonuchal tension, headache, cough, fever and odynophagia. Her past medical history included arterial hypertension, well controlled with Amlodipine and Olmesartan. Approximately 2 weeks before the admission, she developed pharyngotonsillitis and cough complicated by fever treated with ibuprofen without clinical improvement. Subsequently, she also developed a persistent headache, poorly responsive to pharmacological treatment with paracetamol, prompting her admission to emergency department. On arrival, she was pyretic (body temperature 37.9°C) and tachypnoeic (22 breaths/min). The oxygen saturation was 97% on room air. Chest auscultation revealed normal breath sound. Laboratory tests revealed neutrophilic leukocytosis (11.3 × 10^9^/L including neutrophils 9.69 × 10^9^/L and lymphocytes 1.01 × 10^9^/L), markedly elevated inflammatory markers (C‐reactive protein [CRP] 27.5 mg/dL, procalcitonin [PCT] 5.65 ng/mL) and elevated serum D‐dimer (3555 μg/L). An MRI angiogram of head and neck revealed acute sphenoidal sinusopathy and subocclusive thrombosis of the J3 segments of both IJVs. The patient started empiric intravenous antibiotic therapy with ceftriaxone and clindamycin in order to cover aerobic and anaerobic bacteria, due to the suspicion of bacterial sinusopathy complicated by vascular involvement [12]. Three days after admission, the patient exhibited new‐onset severe dyspnea. Given this rapid clinical change and a markedly elevated serum D‐dimer, pulmonary embolism (PE) was suspected. A CT pulmonary angiography (CTPA) confirmed subsegmental pulmonary arterial thromboembolism involving the right lower and upper lobe and lingula (Figure 1). Anticoagulation therapy was started. Additionally, the same imaging revealed 12 solid parenchymal nodules with ill‐defined margins, five of which were cavitated, distributed throughout the lung lobes, along with minimal bilateral pleural effusion (Figure 2).

After 5 days in the emergency department, the patient was transferred to our Pulmonology ward for a suspicion of a primary lung cancer or secondary pulmonary metastases associated with PE. At our department, we performed microbiological tests that came back negative (blood cultures, urinary antigen tests for *Legionella pneumophila* and *Streptococcus pneumoniae* , QuantiFERON TB Gold). A right‐sided infective endocarditis as a potential source of septic pulmonary emboli was ruled out by performing a transthoracic echocardiogram, which showed no abnormalities. Deep vein thrombosis (DVT) was ruled out by colour doppler ultrasound. Thrombophilia and autoimmunity serological panels were negative. Flexible fibrobronchoscopy showed edematous and hyperemic pharyngeal mucosa in the absence of vegetative and/or infiltrative endoluminal bronchial lesions bilaterally. Bronchoalveolar lavage was collected within lingular bronchial subsegments. Cytological and microbiological analysis were negative for infections or malignancy. Microbiological analysis included culture test for common bacteria, *Mycobacterium tuberculosis* and Non‐*Tuberculous mycobacteria* (NTM), *Galactomannan aspergillus*, *Nocardia* spp. and *Pneumocystis carinii*. Considering the bilateral IJV thrombophlebitis, septic pulmonary emboli and a recent history of pharyngeal disease, a diagnosis of LS with concurrent PE was made, according to diagnostic criteria for LS [10]. After 2 weeks of intravenous treatment with ceftriaxone and clindamycin, the patient showed a marked clinical improvement (resolution of fever, pharyngitis and headache) and normalisation of inflammatory markers. She was discharged home on oral clindamycin for a further 4 weeks, in addition to anticoagulant therapy with warfarin. One month follow‐up CT angiography of the neck and chest showed complete resolution of bilateral IJV thrombosis and of pulmonary arterial thromboembolism and a complete regression of pulmonary nodules (Figure 2).

## 3 Discussion

The peculiarity of our case is that, in addition to pulmonary septic emboli, a subsegmental pulmonary arterial thromboembolism detected by CTPA also developed. This radiological dualism, characterised by a thrombotic component in the pulmonary arterial vessels and a septic component of cavitated nodules in the lung parenchyma, is extremely rare in LS. This dual radiological pattern may be explained pathophysiologically by a severe thrombotic burden due to systemic inflammation, endothelial damage and sepsis‐induced hypercoagulability, the so‐called immunothrombosis [13, 14]. In addition, our patient was 65 years old, a higher age than the mean age of LS patients (17–33 years) [3]. The credibility of the diagnosis of LS in our case, even though we did not isolate a microbiological agent, is completely supported by recent evidence on the relaxation of the restriction regarding the diagnostic criteria for LS, especially in patients already on empirical antibiotic therapy, such as our case [15]. Actually, the isolation of Fusobacterium is not considered to be mandatory also due to its difficulty to isolate in blood cultures and to the empiric antibiotic therapy that often has been started before the collection of culture tests, like in our clinical case [7, 10]. The current available data reveal that around 90% of cases isolated the responsible bacterium, with FN present in 68% and 51%, respectively [2, 3]. The difficulty in isolating FN is due to the presence of the bacterium in small numbers in culture broths and the lack of efficient media for selecting the micro‐organism from contaminated samples [16]. The lung is the site where septic emboli most frequently form, ranging from 71% to 91% [3, 5].

The patient was given antibiotic therapy with ceftriaxone and clindamycin in line with recent recommendations, to cover a broad spectrum of bacteria including anaerobes [17, 18]. The patient's clinical and laboratory progress made it possible, after 2 weeks of intravenous antibiotic treatment, to continue this therapy orally at home.

The role of anticoagulant therapy in LS is still controversial today. A recent meta‐analysis shows a usage prevalence of 56% [3]. The main reasons for the current non‐widespread use of anticoagulant therapy is the risk of bleeding and/or rupture of septic thrombi with formation of new emboli. Moreover, there are some retrospective studies that show no effective role of anticoagulant therapy in vessel recanalization during follow‐up, and that thrombosis resolved only with antibiotic therapy [17]. In contrast, other case series indicate a beneficial and supportive role of anticoagulant therapy in the resolution of LS thrombosis [18]. A recent post hoc observational study showed that anticoagulant therapy had no significant impact on the progression or recurrence of thrombotic or septic episodes [19]. In contrast, a recent meta‐analysis showed at multivariate analysis that anticoagulant therapy was a positive prognostic factor [16]. In our clinical case, the presence of pulmonary arterial thromboembolism in addition to IJV thrombophlebitis justified the use of anticoagulant therapy during admission and at discharge home.

The literature review was conducted through the PubMed database. The search terms included: “*Pulmonary thromboembolism*” AND “*Lemierre syndrome*”. A total of 101 articles were obtained, in which only two articles met the inclusion criteria. The inclusion criteria were: 1. Confirmed diagnosis of LS; 2. Radiological evidence of pulmonary septic emboli; 3. Radiological evidence of pulmonary arterial thromboembolism.

The first article is a case report by De Giorgi A et al. describing a 53‐year‐old man who had a one‐month history of fever, occipital headache and chest pain [20]. The visible radiological triad included septic pulmonary emboli, pulmonary arterial thromboembolism and IJV thrombosis. All microbiological cultures were negative. The patient received anticoagulant therapy with low molecular weight heparin and antibiotic treatment with levofloxacin. Radiological follow‐up at 1 month showed complete IJV recanalisation.

The second article is a case report by Jassal SS et al. [21], describing an 18‐year‐old woman with right‐sided pleuritic chest pain and dyspnea arising from 12 h. Her laboratory tests revealed acute renal failure and elevation of inflammatory markers, including D‐dimer. A ventilation‐perfusion lung scintigraphy (V/Q scan) revealed a subsegmental pulmonary arterial thrombus while chest CT revealed a right pleural empyema requiring thoracic drainage. Post‐drainage chest CT showed multiple septic pulmonary emboli in the right lung. Pleural fluid culture was positive for FN, confirming the diagnosis of LS. The patient was treated with meropenem plus metronidazole during hospitalisation (followed by amoxicillin‐clavulanate upon discharge) and anticoagulant therapy with enoxaparin. One month follow‐up imaging showed a near‐complete resolution of the pleural effusion. A summary table of this literature review can be consulted at Table 1.

**TABLE 1:** Pulmonary arterial thromboembolism in Lemierre's syndrome: Summary table of the literature review.

Case report	Age, years	Sex	Symptoms	Bacteria isolated	Radiological findings	Antibiotic therapy	Anticoagulant therapy
Fabozzi A et al.	65	F	Occipital headache Fever Cough Odynophagia	None	1. IJV thrombophlebitis 2. Septic pulmonary emboli 3. Pulmonary arterial thromboembolism	Ceftriaxone Clindamycin	Enoxaparin (in hospital) Warfarin (at home)
De Giorgi A et al. [20]	53	M	Occipital headache Fever Chest pain	None	1. IJV thrombophlebitis 2. Septic pulmonary emboli 3. Pulmonary arterial thromboembolism	Levofloxacin	Enoxaparin
Jassal SS et al. [21]	18	F	Pleuritic chest pain Dyspnea	FN	1. Septic pulmonary emboli 2. Pleural empyema 3. Pulmonary arterial thromboembolism	Meropenem Metronidazole	Enoxaparin

Abbreviations: FN = *Fusobacterium necrophorum* ; IJV = internal jugular vein.

## Author Contributions

A.F., A.S. and S.I. wrote the manuscript; A.S., M.B. and P.P. reviewed the manuscript.

## Consent

The authors declare that written informed consent was obtained for the publication of this manuscript and accompanying images using the form provided by the Journal.

## Conflicts of Interest

The authors declare no conflicts of interest.

## Data Availability

Data sharing is not applicable to this article as no new data were created or analyzed in this study.
